# Boston School of Medical Specialties

**Published:** 1868-02

**Authors:** 


### BOSTON SCHOOL OF MEDICAL SPECIALTIES.

We are in receipt of a circular from Dr. Storer, announcing the organization of a corps of teaches for "Special Instruction in Medial Science." A notice of this, the first institution of the kind in America, was intended for the December number of this Journal, but was unavoidably crowded out. The curriculum embraces twelve departments of special subjects, not usually taught in Medical Colleges, nor is it possible to do so in the time allowed, without neglect of the fundemental principles of science. "Diseases of the skin," "Operative ophthalmology," "Sugical deformities" and "Venerial disease," are some of the special subjects taught in the school.

